# Moonlighting chromatin: when DNA escapes nuclear control

**DOI:** 10.1038/s41418-023-01124-1

**Published:** 2023-02-08

**Authors:** Jeeshan Singh, Michael Boettcher, Maximilian Dölling, Annika Heuer, Bettina Hohberger, Moritz Leppkes, Elisabeth Naschberger, Mirco Schapher, Christine Schauer, Janina Schoen, Michael Stürzl, Ljubomir Vitkov, Han Wang, Leticija Zlatar, Georg A. Schett, David S. Pisetsky, Ming-Lin Liu, Martin Herrmann, Jasmin Knopf

**Affiliations:** 1 Department of Internal Medicine 3, Rheumatology and Immunology, Friedrich-Alexander-Universität Erlangen-Nürnberg (FAU) and Universitätsklinikum Erlangen, Erlangen, Germany; 2 Deutsches Zentrum für Immuntherapie (DZI), Friedrich-Alexander-Universität Erlangen-Nürnberg (FAU) and Universitätsklinikum Erlangen, Erlangen, Germany; 3 Department of Pediatric Surgery, University Medical Center Mannheim, University of Heidelberg, Mannheim, Germany; 4 Department of Surgery, University Hospital Magdeburg, Magdeburg, Germany; 5 Division of Spine Surgery, Department of Trauma and Orthopedic Surgery, University Medical Center Hamburg-Eppendorf (UKE), Hamburg, Germany; 6 Mildred-Scheel Cancer Career Center Hamburg HaTriCS4, University Cancer Center Hamburg, Hamburg, Germany; 7 Department of Ophthalmology, Friedrich-Alexander-Universität Erlangen-Nürnberg (FAU) and Universitätsklinikum Erlangen, Erlangen, Germany; 8 Department of Internal Medicine 1, Gastroenterology, Friedrich-Alexander-Universität Erlangen-Nürnberg (FAU) and Universitätsklinikum Erlangen, Erlangen, Germany; 9 Division of Molecular and Experimental Surgery, Universitätsklinikum Erlangen, Friedrich-Alexander Universtität Erlangen-Nürnberg (FAU), Erlangen, Germany; 10 Comprehensive Cancer Center Erlangen-EMN, Universitätsklinikum Erlangen, Erlangen, Germany; 11 Department of Otorhinolaryngology, Head and Neck Surgery, Friedrich-Alexander University Erlangen-Nürnberg (FAU) and Universitätsklinikum Erlangen, Erlangen, Germany; 12 Department of Otorhinolaryngology, Head and Neck Surgery, Paracelsus University, Nürnberg, Germany; 13 Clinic of Operative Dentistry, Periodontology and Preventive Dentistry, Saarland University, Homburg, Germany; 14 Department of Environment & Biodiversity, University of Salzburg, Salzburg, 5020 Austria; 15 Department of Dental Pathology, University of East Sarajevo, East Sarajevo, Republic of Srpska Bosnia and Herzegovina; 16 Department of Medicine and Immunology and Medical Research Service, Duke University Medical Center and Veterans Administration Medical Center, Durham, NC USA; 17 Department of Dermatology, Perelman School of Medicine, University of Pennsylvania, Philadelphia, PA 19104 USA; 18 Corporal Michael J. Crescenz VAMC, Philadelphia, PA 19104 USA

**Keywords:** Inflammation, Cell death and immune response

## Abstract

Extracellular chromatin, for example in the form of neutrophil extracellular traps (NETs), is an important element that propels the pathological progression of a plethora of diseases. DNA drives the interferon system, serves as autoantigen, and forms the extracellular scaffold for proteins of the innate immune system. An insufficient clearance of extruded chromatin after the release of DNA from the nucleus into the extracellular milieu can perform a secret task of moonlighting in immune-inflammatory and occlusive disorders. Here, we discuss (I) the cellular events involved in the extracellular release of chromatin and NET formation, (II) the devastating consequence of a dysregulated NET formation, and (III) the imbalance between NET formation and clearance. We include the role of NET formation in the occlusion of vessels and ducts, in lung disease, in autoimmune diseases, in chronic oral disorders, in cancer, in the formation of adhesions, and in traumatic spinal cord injury. To develop effective therapies, it is of utmost importance to target pathways that cause decondensation of chromatin during exaggerated NET formation and aggregation. Alternatively, therapies that support the clearance of extracellular chromatin are conceivable.

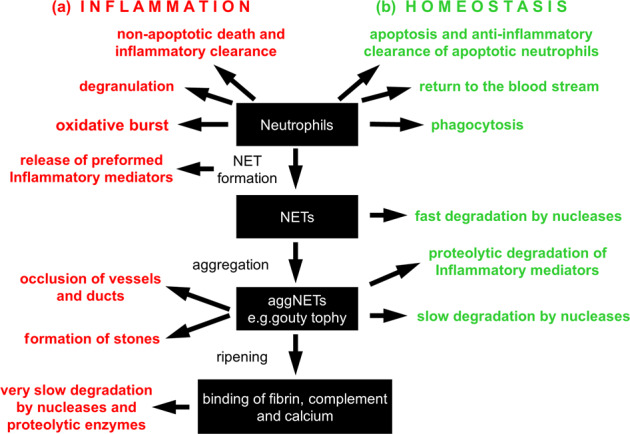

## Facts

- DNA has two jobs. DNA is (I) responsible for heredity, and (II) can moonlight to orchestrate certain pathways of the immune response.
- The pro-inflammatory/pro-thrombotic activity of extracellular DNA can be driven by (aggregated) neutrophil extracellular traps (NETs).
- Rupture of the nuclear envelope, chromatin decondensation, loading of the chromatin with granular and cytoplasmic proteins, and plasma membrane breakdown are key cellular events for the release of chromatin during NET formation.
- Reactive oxygen species (ROS) and an increase of intracellular calcium levels activate several downstream effectors that are crucial for NET formation.
- Myeloperoxidase (MPO), neutrophil elastase (NE), peptidyl arginine deiminase 4 (PAD4), and a plethora of further intracellular proteins determine the functional capabilities of NETs.
- Extracellular chromatin’s moonlighting tasks foster various pathological conditions and drive inflammatory diseases such as spinal cord trauma, cancer, sepsis, immunothrombosis, periodontitis, obstruction of exocrine ducts and glands, and formation of stones or adhesions.

## Open questions

- How can unbalanced actions of extranuclear DNA-protein complexes be rebalanced?
- What therapies can effectively block pathways leading to aberrant NET formation?
- What therapies can support the clearance of NETs and their aggregates?
- How can the protective role of extracellular chromatin be preserved?

## Introduction

DNA is a polymeric macromolecule that displays distinct molecular and functional properties depending on its location [1–3]. Inside the nucleus, DNA serves as the essential molecule of heredity, encoding information for gene structure and regulation. Nuclear DNA is bound to histones in the form of nucleosomes, constituting a material known as chromatin [4, 5]. Once outside the cell, DNA can expand in space and display other functional activities to drive inflammation and thrombosis [6]. Moonlighting is an extra activity or occupation, sometimes performed in secret. If heredity and gene regulation are DNA’s main functions in the nucleus, immunity is DNA’s main function in the extracellular space, whether tissue or blood.

The structural bases of the intracellular and extracellular activities of DNA differ. The nuclear functions of DNA result from gene sequences and base modifications, while the extracellular functions result primarily from the charged phosphodiester backbone and its extended polymeric structure. The structures of DNA, both sequence and backbone, facilitate the binding of proteins and provide the basis for a multitude of intermolecular interactions. The translocation of DNA from the inside to the outside of the cell is the key mechanism that reveals the full diversity of DNA’s biological activities [5].

As demonstrated in many model systems, the translocation of DNA outside the cell can occur with cell death, stress and injury, with cell death being the predominant source of extracellular DNA [7–9]. With cell death, DNA is a byproduct that is often considered as debris. This DNA is subject to rapid removal. With persistence and heightened levels, however, DNA can become noxious or “dangerous” as it can enter cells and interact with nucleic acid sensors; these sensors are part of an internal host defense system which can be triggered by foreign DNA from bacteria or viruses as well as self-DNA arising from cell stress or impaired nuclease activity [10, 11].

In addition to inadvertent or programmed cell death, extracellular DNA and chromatin can occur in the context of neutrophil extracellular trap (NET) formation [12]. NET formation is an elaborate program of polymorphonuclear granulocytes that involves the movement of DNA from the nucleus to the cytoplasm where it mixes with the contents of granules to form NETs. The latter play diverse and important roles in inflammation. The principal components of NETs, DNA and histones, are ancient and can even be found in archaea. From the point of view of evolution, it is noteworthy that extracellular chromatin decorated with histones and other antimicrobial proteins also occurs in invertebrates such as crabs, mussels and sea anemones [13], fish [14, 15], birds [16], as well as protozoans and plants [17].

Also, in mammals, extracellular DNA traps can originate not only from neutrophils [18] but also from other immune cells (eosinophils, dendritic cells, monocytes, macrophages, mast cells, basophils, T cells, and B cells); DNA traps can also arise from non-immune cells (endothelial cells, platelets, and cardiomyocytes) [19]. The evolutionary conservation of DNA traps suggests that the evolution of DNA has involved both hereditary and gene regulation as well as the potential weaponization against invading pathogens [20].

Depending on whether extracellular DNA or chromatin arises from cell death or NET formation, the molecular properties of the DNA, as well as the identity of associated macromolecules (e.g., histones, enzymes) will differ. The release of DNA from dead and dying cells can be studied in both in vitro as well as in vivo models, although in vivo models allow better assessment of potential mechanisms of clearance and degradation as well as interplay of dead cells with phagocytic cells [21–24]. In vivo models to study DNA translocation can involve the transfer of dead and dying cells to a recipient animal or the in vivo induction of apoptosis or necrosis. Other models involve infection or the stimulation of inflammation that can lead to cell death as well as NET formation. In models tested thus far, extracellular DNA shows a major peak of approximately 166 bases – the size of a mononucleosome – no matter whether induction of death was by apoptosis or necroptosis [21, 22]. This size range is the same as that observed in studies on the molecular properties of DNA in the blood [25].

As demonstrated in in vivo models, the translocation of DNA into the blood depends on macrophages and can be modulated by glucocorticoids as well as sex hormones [22, 26–28]. Thus, the occurrence of DNA in the blood is the culmination of complex processes that are subject to strict regulation, including nucleolytic digestion. As these processes proceed, the size of extracellular DNA changes since DNA, when released during NET formation, for example, can show very high molecular weight (thousands to tens of thousands of bases) while, in the blood, most of the DNA is less than 200 bases [25].

In addition to a soluble form, extracellular DNA can exist as a particle [29]. This particulate form of DNA resides in microparticles which are released from cells during apoptosis and likely correspond to blebs on the cell surfaces [30, 31]. This DNA is accessible and antigenically active and can be bound by monoclonal anti-DNA antibodies as well as sera from patients with systemic lupus erythematosus (SLE) to form large immune complexes [32–36]. Mitochondria represent a further source for extracellular DNA in a particle form that can bind anti-DNA antibodies [37]. Recognition of the various physicochemical forms of DNA circulating in the blood is important since their detection may differ depending on the use of sera or plasma as well as the conditions for isolation and analysis.

In this conceptualization, extracellular DNA or chromatin is an ensemble of molecules that vary in their origin from different cell populations; mechanisms of translocation (e.g., apoptosis, necrosis, NET formation); different physicochemical forms (i.e., high vs low molecular weight, soluble vs particulate); and the array of associated macromolecules. Rather than debris or a simple byproduct of cell death, extracellular DNA represents a multifunctional complex that displays activities to drive the pathogenesis of many diseases. Importantly, extracellular DNA and chromatin can provide a structure to organize and promote the activity of other mediators and thereby intensify inflammation and drive thrombosis.

The pro-inflammatory and pro-thrombotic activity of extracellular DNA occurs prominently during the process of NET formation, a unique element in host defense based on the elaboration of extracellular DNA in a high molecular weight form that can serve as a scaffold decorated by other intracellular molecules. This review will focus on the extracellular release of DNA during the process of NET formation and the many roles that NETs can play in disease.

## The process of NET formation and degradation

**Fig. 1:**
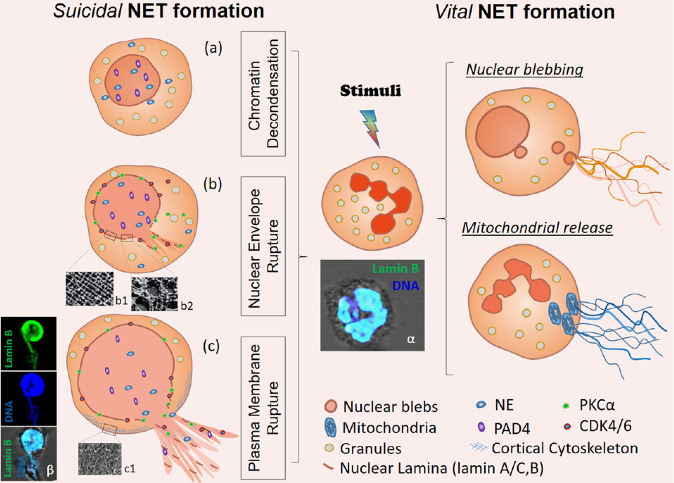
Mechanisms of Neutrophil NET formation. Suicidal NET formation: **a** Chromatin decondensation is mediated by PAD4 and/or NE. **b** Nuclear envelope rupture is modulated by nuclear translocation of PKCα or CDK4/6 which mediate nuclear lamina disassembly (electron microscopy images of b1 well organized nuclear lamina, or b2 disassembled nuclear lamina [256]). **c** Rupture of the plasma membrane is achieved by disassembly of cortical cytoskeleton (c1, electron microscopy image of actin cortex [257]). (α, β) Representative confocal microscopy images of an untreated neutrophil (α) and a PMA-treated neutrophil with ruptured nuclear envelope and extracellular NETs in which nuclear DNA forms the backbone of NETs that are decorated with the disassembled lamin B (β), stainings of lamin B and DNA with fluorescent-labeled anti-lamin B1 and DAPI. Vital NET formation: Vital NET formation has been described as either derived through nuclear blebbing, or released from mitochondria.

Depending on the cellular viability, NET formation has been classified as suicidal or vital NET formation [38–42]. Since the nuclear genome (~3.2 billion bp) is 200,000 times larger than the mitochondrial genome (16,569 bp) [38], nuclear DNA can indisputably form the backbone of the structure of NETs in suicidal NET formation (Fig. 1) [43]. However, mitochondrial DNA and over 20 other components are also associated with NETs [44, 45]. The nucleus is the source for extracellular DNA NETs in suicidal NET formation. In the nucleus, chromatin is enclosed by the nuclear envelope, which consists of outer and inner nuclear lipid membranes (ONM and INM) and the underneath nuclear lamina [38, 43, 46]. The latter is a filamentous structure consisting of A-type (A, C) or B-type (B_1_, B_2_) lamins [46]. A-type lamins are assembled as thick filament bundles, which affect the mechanical properties of the nuclei. In contrast, B-type lamins are assembled as a thin but highly organized meshwork which is crucial to the integrity and elasticity of the nuclear envelope [38, 43, 46].

The nuclear envelope is the first physical barrier for chromatin extranuclear extrusion. Nuclear envelope rupture occurs when the nuclear lamina is either cleaved proteolytically or disassembled by phosphorylation [38] (Fig. 1b). Lamin B is proteolytically cleaved by caspase-3 during apoptosis [43]. However, NET formation is a caspase-independent process [43, 47, 48], during which caspase-3 remains inactive [43]. Recent studies indicate that protein kinase C-α (PKC-α)-mediated lamin B phosphorylation and disassembly is responsible for nuclear envelope rupture [43, 49]. In addition, cyclin dependent kinase 4/6 (CDK4/6) controls NET formation through modulation of lamin A/C phosphorylation, resulting in nuclear envelope rupture [38, 50]. Mice with deficiency of CDK4/6 or PKCα [50, 51], or overexpression of lamin B [43], display impaired NET formation in vivo. Thus, kinase-mediated nuclear lamina phosphorylation-disassembly [43, 50], but not proteolytic cleavage [38], is responsible for nuclear envelope rupture during NET formation. PKCα and CDK4/6 are located in the cytoplasm of resting neutrophils, and their nuclear translocation requires a functional actin cytoskeleton in the early stage of neutrophil activation [38, 49, 52]. Genetic [49, 53] or pharmacologic [54] inhibition of actin assembly or its upstream regulatory molecules, Rho kinase [49] or Wiskott–Aldrich syndrome protein [53], impair NET formation. This argues for a crucial role of the actin cytoskeleton in NET formation [38, 52].

Nuclear DNA is tightly packaged as chromatin by histones. The extranuclear extrusion of chromatin requires its decondensation, which is mediated through histone citrullination by peptidyl arginine deiminase 4 (PAD4) [55] and/or histone cleavage by neutrophil elastase (NE) [39, 47] (Fig. 1a). In resting neutrophils, both PAD4 and NE are located in cytoplasmic granules [56, 57]. PAD4 has a nuclear localization sequence (NLS) which mediates nuclear translocation of cytoplasmic PAD4 [38]. Since NE does not have a NLS, it is unclear how NE is imported into the nucleus [38], however, the actin cytoskeleton might be involved in nuclear translocation of NE [58]. Also, CDK4/6- or PKCα-mediated nuclear envelope rupture may contribute to NE nuclear translocation which can be blocked by inhibition of these kinases [38, 50]. Furthermore, gasdermin D (GSDMD) pores may be involved in NE release from granules and its nuclear translocation. NE in turn may also process GSDMD for its maturation and pore formation in nuclear, granular, and plasma membranes [59, 60].

The plasma membrane is the second physical barrier for extracellular release of nuclear DNA. The cortical actin cytoskeleton is attached underneath the plasma membrane and strengthens its integrity [61, 62]. A recent study found that dynamic changes of actin polymerization in early stage, and actin depolymerization in late-stage, are accompanied by corresponding changes of Rho kinase activities [49]. The aforementioned dynamic changes explain the role of the actin cytoskeleton in the early-stage nuclear translocation of lamin kinase PKCα and CDK4/6 [38, 49, 52], and involvement of actin depolymerization in plasma membrane rupture in later stages of NET formation (Fig. 1c) [49, 62]. Disassembly of cortical cytoskeleton weakens the plasma membrane. This together with expanding forces from chromatin swelling [54], contributes to plasma membrane rupture and extracellular NET release.

Based on emerging evidence [38, 43, 49, 50, 54, 62], rupture of the nuclear envelope, nuclear chromatin decondensation, and the plasma membrane breakdown, are the key and necessary cellular events for nuclear chromatin extracellular release in suicidal NET formation (Fig. 1). The signaling pathways that regulate key cellular morphological changes might be candidate targets for therapeutics in NET-related diseases. Since NET formation has been described [63], and detailed in seminal experiments [18], the involvement of various signaling pathways has been reported [12, 38, 39]. Reactive oxygen species (ROS) are crucial for NET formation as they activate several downstream effectors. ROS modulate the release of granule myeloperoxidase (MPO) and NE [39, 47], and regulate cytoskeletal dynamics [53], which is involved in NET formation [49, 53, 54, 62]. Activated neutrophils generate ROS through activation of nicotinamide adenine dinucleotide phosphate (NADPH) oxidase-2 (NOX2) or mitochondrial dysfunction [39, 47, 48, 64]. Depending on the stimuli, NOX-dependent, and -independent pathways have been reported to drive NET formation [39, 47, 48, 64].

In NOX-dependent pathways, stimuli (like, PMA, LPS, PAF) activate NOX2 that drives NET formation through ROS generation [39, 47, 48, 65], while genetic mutation or pharmacological inhibition of NOX2 attenuates NET formation [39, 47, 48]. However, requirements for NOX are stimulus dependent, and NOX activity is not required for calcium ionophore-induced NET formation [66]. Calcium ionophores induce calcium influx, which activates the mitochondrial SK3 channel, resulting in mitochondrial ROS production for NOX-independent NET formation [64]. A recent study found that calcium ionophores activate calpain, which may with involvement of calcium-dependent PAD4 mediate interdomain proteolysis of the nuclear lamina and high mobility group box 1 protein (HMGB1); the latter an architectural chromatin binding protein [67]. The collective activity of PAD4 and calpain may contribute to the destruction of the nuclear lamina and, thus enable chromatin decondensation in calcium-mediated NET formation [67]. However, more detailed studies are needed for understanding the role of calpain in nuclear lamina disintegration. Intracellular calcium mobilization is also involved in regulation of actin cytoskeleton dynamics. These are important in nuclear translocation of PKCα and CDK4/6 for nuclear lamina disassembly [52] and NE for chromatin decondensation [58].

In contrast to suicidal NET formation, “vital NET formation” has also been reported as extracellular release of either mitochondrial [40] or nuclear [41] DNA, without loss of plasma membrane integrity (Fig. 1). Vital NET formation was initially described in neutrophils that were first primed by GM-CSF and then consequently stimulated with LPS/C5a, resulting in rapid release of NETs which DNA is solely from mitochondria [40]. An intact cytoskeleton is required for vital NET formation with involvement of ROS [68]. Extracellular release of mitochondrial DNA from viable cells has been observed not only in granulocytes [68], but also in lymphocytes [69] and amoebae [70]. This phenomenon has been considered an intrinsic innate immune response by either directly killing bacteria [70], or indirectly by inducing anti-viral interferons [68, 69]. Interestingly, mitochondrial DNA release has also been observed in viable fibroblasts [71] or chondrocytes [72], which might be an acute-phase response to mitochondrial stress or dysfunction. The latter findings, however, raise the question if extracellular release of mitochondrial DNA from viable cells is a broader phenomenon not limited to immune cells [73]. More studies are needed to understand this important and interesting phenomenon comparing extracellular release of mitochondrial DNA from viable immune cells vs non-immune cells.

In addition to mitochondrial DNA release, another study reported that exposure of neutrophils to *Staphylococcus aureus* can induce rapid NET formation without cell membrane breakdown [41]. Upon stimulation, the multilobular nucleus rapidly became rounded, followed by nuclear blebbing of chromatin containing vesicles, which deliver and release their contents into the extracellular space for NET formation [41]. The entire process occurs in 5–60 min in a ROS-independent manner [41] and requires chromatin decondensation [74]. Although the mechanism that regulates the nuclear blebbing in vital NET formation is unclear, histone modification during chromatin decondensation may contribute to nuclear blebbing, known to be determined by alteration of chromatin compaction and histone modification [75]. The budded nuclear vesicles may rupture over time and release their enclosed DNA into the cytoplasm, and eventually extrude into the extracellular space as described for suicidal NET formation [41]. Two studies found that parasites may induce rapid vital NET formation at 10–30 mins of neutrophil-parasite interaction, and suicidal NET formation when they are co-incubated for a longer time. The latter condition results in increased total NET formation [74, 76]. One may speculate that vital/rapid NET formation might be the early event of suicidal NET formation before the neutrophils lose their viability. More studies are needed to address the relationship between vital and suicidal NET formation with extracellular release of chromatin.

All in all, the coexistence between the suicidal lytic and vital NET formation remains uncertain [38, 45]. Most importantly, the diversity of NET formation signaling pathways makes it difficult to identify unified targets for therapeutic purposes of NET-related diseases. To control NET formation, there is a need for identifying the pathways in different clinical settings to allow specific targeting. Another option is to target and improve the degradation of NETs for a fine-tuning of the balance between NET formation and degradation. NETs are reportedly degraded by macrophages; preprocessing of NETs with DNase1 and opsonization with C1q facilitates this process [77]. Macrophages take up NETs through micropinocytosis, but how exactly the degradation is achieved warrants further research [77, 78]. Dendritic cells (DCs) are able to take up NETs, albeit to a much lesser extent than macrophages, and to secrete DNase1L3 for extracellular digestion of NETs [78]. Recently, it was also described that 13-series (T-series) resolvins reduce NET formation by enhancing NET uptake by human macrophages in a phospho–AMP-activated protein kinase (AMPK)-dependent manner [79]. One should be cautious in enhancing NET degradation since degraded NETs reportedly foster the growth of *Actinobacillus pleuropneumoniae*, causing severe porcine pneumonia;[80] this might also be true for further pathogens. The degradation of NETs by circulating DNases leads to the release of so-called NET degradation products (NDPs) such as cell-free DNA (complexed with MPO or NE) and histones. These NDPs themselves have toxic effects like fixation of complement (cell-free DNA) [81], induction of oxidative tissue damage (MPO) [82], promotion of thrombosis by local proteolysis of the tissue factor pathway inhibitor [83], or activation of platelets and cytotoxicity for epithelial cells (histones) [84–87].

## The composition of NETs

### NET-borne enzymes

During the process of NET formation or cell death, chromatin escapes the nuclear control with an abundance of various proteins. In a first assessment of the neutrophil proteome, a total of 251 major cellular proteins in different compartments were identified by gel-LC-MS/MS [88]. This proteome included the azurophilic granule proteins NE and MPO as well as PAD4, which catalyzes the deimination of arginine to citrulline and mediates chromatin decondensation [89, 90, 55]. In a recent proteome analysis by Petretto et al., 330 NET-associated proteins were identified; many with posttranslational modifications [91]. Of these, 74 were detected in all NETs but others differed dependent on the inducer of NET formation. Interestingly, the cellular origin of these NET-associated proteins seemed independent of the respective inducers with most proteins originating from the cytoplasm/cytoskeleton, followed by organelle- and membrane-derived proteins. The identification of only four to six different proteins associated with NETs from patients with SLE compared to rheumatoid arthritis (RA) determined that the nature of the stimulant is more important for the NET proteome composition than the underlying disease profile [92].

### Aggregation of NETs

**Fig. 2:**
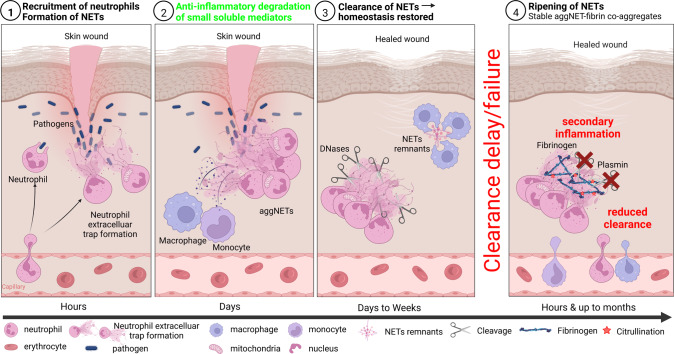
Illustrative representation of the neutrophil extracellular trap (NET) formation and degradation cascade. 1. Neutrophils, via a trans-endothelial mechanism, are recruited to the site of an incident within hours. There they form NETs by releasing nuclear chromatin or mitochondrial DNA decorated with potent antimicrobial granular proteins. 2. Monocyte and activated macrophages secrete neutrophil chemoattractants that lead to a rapid influx of a large number of neutrophils. At these high densities, neutrophils aggregate, forming so-called aggregated NETs (aggNETs). These foster the resolution of inflammation by degrading small soluble mediators of inflammation. 3. Within days to weeks, after NETs enabled a successful entrapment of pathogens and restricted their dissemination, intracellular DNases break down the DNA backbone of NETs. These remnants can then be cleared by phagocytic cells (e.g., macrophages) to promote clearance and thereby restore homeostasis. 4. Ineffective clearance of NETs leads to their extended and prolonged ripening by forming stable aggNET-fibrin co-aggregates, in which fibrin polymerizes in the scaffold formed by NETs. In these aggregates fibrin can be citrullinated and, consequently, resists degradation by plasmin. In addition, the NETs incorporated in these aggregates were protected from DNases. A reduced clearance then may lead to long-term secondary inflammation and formation of stones and tophi.

The binding of NE and other antimicrobial proteins to extruded chromatin of NETs mediate the digestion and elimination of microbial pathogens at the site of insult. At these areas of high cell densities, neutrophils tend to aggregate and form enzymatically stable clumps called aggregated NETs or aggNETs [93]. NETs and aggNETs immobilize, neutralize and/or kill bacteria [18], fungi [94], viruses [95], parasites [96], and inhibit their dissemination [97–99]. In addition, aggNETs also contribute to the resolution of inflammation. The externalized chromatin fibers are decorated with a plethora of cytoplasmic and granular proteases. Consequently, aggNETs can not only scavenge inflammatory mediators, but also degrade these molecules [100]. The proteolysis of toxic molecules like highly cationic histones, which had entered the extracellular space during NET formation, protects surrounding tissues from chronic damage and allows re-establishment of tissue homeostasis [101]. AggNETs also sequester and degrade inflammatory cytokines and chemokines. This process prevents further recruitment of neutrophils and supports the resolution of inflammation [93]. Despite possessing beneficial effects, NETs are also involved in the induction of pro-thrombotic events. NET-bound histones interact directly with T-cells resulting in Th17 differentiation [102], and activate platelets, thereby stimulating thrombogenesis [103]. Impaired NET aggregation and clearance can drive the development of autoimmunity and become detrimental. Therefore, a dysregulated immune response and an imbalance between NET formation and degradation can lead to devastating diseases as summarized in Fig. 2 and in the following paragraphs.

## Methods to detect NETs in tissue samples

The detection of tissue-borne NETs is crucial to identify dysregulated NET formation and clearance in infections, sepsis, autoimmune diseases, thrombosis, metabolic disorders, and cancer. Currently, the most general technique to visualize NETs in tissues is immunostaining of paraffin-embedded tissues followed by immunofluorescence microscopy. After deparaffinization of tissue sections, heat-induced epitope retrieval (HIER) buffer is used for rehydration, breaking the methylene bridges and making epitopes such as NE, MPO, and citrullinated histone H3 accessible for binding of antibodies. The antibody-stained tissues are counterstained with DNA intercalating dyes that have detected extended NETs in many tissue sections with reliable signal intensity [104]. Recently, a protocol for multiplex staining demonstrated the detection of NETs in paraffin-embedded human biopsies of phlegmonous appendicitis, lung abscess and non-small cell lung cancer [105]. Over the past two decades, numerous technological advances have been made in immunofluorescence microscopy. Recent achievements allow greater insights into the morphology and high-resolution analysis to detect subtle changes in the tissues. High resolution stimulated emission depletion (STED) microscopy was used to detect citrullinated NETs frequently recurring in tissue biopsies from patients with colon cancer. Using anti-DNA antibodies directed to extracellular chromatin, the authors distinguished between condensed and compacted chromatin inside neutrophils with a healthy appearance and ejected decondensed DNA displaying the typical characteristic of NETs [106].

Label-free emission recorded in immunofluorescence microscopy of tissue biopsies from patients with COVID-19 revealed widespread immune thrombosis in a devastated pulmonary vasculature due to native endogenous fluorophores. Contrary to standard detection of NETs via immunofluorescence using antibodies, a recently published article reported the detection of NETs in inflammatory diseases using a fluorogenic peptide. The authors developed a highly specific, triple-quenched, tri-branched fluorescent human neutrophil elastase sensor. This sensor offers a more than 20-fold increase in fluorescence intensity upon enzymatic cleavage and extends the current detection methods from antibodies to the first molecular probe detectors [107].

Additionally, several live-cell imaging techniques directly monitor the neutrophils’ release of NETs at the cellular level in various complex tissue and organs affected by infection and autoimmunity. Two-photon microscopy enables real-time detection of NETs in *Aspergillus fumigatus* infected murine lung lobules using the SYTOX dye without organ fixation [108]. Intravital microscopy showed activated platelets resulting in the formation of NETs in liver sinusoids [109], and using SYTOX dye, spinning disk confocal intravital microscopy visualized the formation of NETs, over two hours on murine skin infected with *Staphylococcus aureus* [110–112]. Cell-permeable and impermeable DNA dyes allow the direct visualization of intact neutrophils and of NETs in various organs, respectively [112–114]. Intravital microscopy with a laser scanning microscope characterized NETs in blood vessels of different organs [110, 115].

To date, classical antibody-based immunofluorescence methods are still the most commonly used to detect neutrophils and NETs in tissue sections. However, many promising new methods like live-cell imaging and STED microscopy have recently been developed to directly monitor neutrophils and NETs in vivo and with ultrahigh resolution, respectively.

## Extracellular chromatin moonlighting diseases

### Immunothrombosis

In the last decade, it has become increasingly clear that neutrophils and especially NETs are intertwined into the processes of thrombus formation and maturation in diverse pathological settings [116]. The term immunothrombosis has been coined to highlight the interaction of the cellular innate immune system with pathological thrombosis [117]. The evolutionary advantage of the activation of thrombosis by players of the innate immune system lies in physically trapping infectious agents in occluded vessels to limit spread via the circulation and thus contain an inflammatory focus [118]. Exaggerated immunothrombosis, however, is central in the exacerbation of several pathological settings including coagulopathy in sepsis [109], necroinflammation [119], and severe COVID-19 [120, 121]. As shown in pancreatitis [122], not only blood vessels, but also glandular ducts can be occluded by NETs. Apart from physical trapping of microbes, immunothrombosis also fulfils beneficial hemostatic tasks in the setting of mucosal damage in acute flares of ulcerative colitis [123]. Here, the absence of PAD4 is associated with increased mucosal blood loss. The interactions of neutrophils and components of NETs with classical players of thrombosis are numerous; these players include platelets, serine proteases of the coagulation cascade, fibrinolysis and the fibrin mesh itself. Neutrophils and NETs interact with these players via membrane-bound receptors, degranulated effector proteins, chromatin of NETs, including NET-bound nuclear and granular proteins [124] and extracellular vesicles [125]. Activated platelets can induce formation of NETs [126]. More specifically, deletion of HMGB1 in platelets reduced NET formation and associated organ damage in various experimental models [127]. Vice versa, platelets may bind to and aggregate on extracellular chromatin of NETs [103].

The aggNETs provide a scaffold for thrombus formation and are able to occlude vessels and ducts [103, 128]. The obstruction of the microvasculature in organs and a consequent inhibition of the blood flow in the capillaries, together with NET-driven endothelial dysfunction, may precipitate organ failure and mortality [129, 130]. Thereby, aggNETs can contribute to the pathogenesis of various diseases. As already mentioned above, NETs occlude the vessels in patients with COVID-19 [120]. Furthermore, NETs-associated occlusions have been reported for coronary vessels in acute myocardial infarction and artherosclerosis [131, 132] and for cerebral vessels in ischemic stroke [133].

In addition to cellular interactions which may foster NET formation and pathological thrombosis, soluble mediators are studied. PAD4 becomes a focus of attention since it reportedly links inflammation and thrombosis. Injection of recombinant human PAD4 in vivo induced the formation of von Willebrand factor (vWF)-platelet strings in mesenteric venules. These strings are naturally degraded by ADAMTS13, a metalloproteinase, but citrullination of ADAMTS13 dramatically reduces the endogenous enzymatic activity [134]. In line with these findings, studies have shown that a class of serpins with an arginine residue in the P1 position (including antithrombin, C1INH, 1-antiplasmin, PAI1/PAI2) is inhibited by citrullination [135, 136], thus unleashing the proteolytic power of the serine proteases thrombin, plasmin and tissue plasminogen activator in the thrombo-inflammatory microenvironment. PAD4-guided thrombin activation may then further facilitate thrombus maturation by FXIII-mediated cross-linking [123]. Citrullination has also recently been identified as a major posttranslational modifier that impacts proteolysis [67]. Future studies will unravel the translational potential of targeting immunothrombosis in clinical settings. These studies could explain the puzzling failure of classical anticoagulants to prevent thrombus formation under certain septic conditions and in disseminated intravascular coagulopathy. Pan-PAD inhibitors and PAD4-specific inhibitors are already central tools in the research of NETs and immunothrombosis and will surely be further studied as therapeutic options in general.

### NETs in lung diseases

Chronic respiratory diseases affect the airways and other structures of the lung [137]. In 2017, 544.9 million people worldwide were affected by a chronic lung disease, such as asthma or chronic obstructive pulmonary disease (COPD), making them the third leading cause of death behind cardiovascular diseases and cancer [138]. Even though asthma was always considered to be an eosinophilic disease, recent reports also highlight the role of neutrophils in this disease [139–141]. Similar to this subset of patients with neutrophilic asthma, patients with COPD show high neutrophilic airway inflammation; higher levels of blood neutrophil counts have been correlated with mortality in these patients [142, 143]. In both lung diseases, NETs were found in the airways of patients and were associated with inflammation [144], and in the case of COPD, also with airflow limitation [145].

Next to chronic respiratory diseases, acute lung injury and acute respiratory distress syndrome (ARDS) are further major causes of morbidity and mortality, especially in the critically ill patients. In these disorders, acute lung inflammation, as indicated by excessive transepithelial neutrophil migration and the release of pro-inflammatory and cytotoxic mediators, disrupts the endothelial and epithelial barriers of the lungs [146, 147]. Increased plasma levels of NETs have been associated with ARDS severity and mortality and lower plasma levels of DNase1 were associated with the development of sepsis-induced ARDS. This indicates that a balance in NET formation and degradation is crucial to prevent lung injury [148]. In this context, disulfiram, an aldehyde dehydrogenase inhibitor, was recently shown to inhibit NET formation and to protect from acute lung injury in a mouse model [149].

Cystic fibrosis (CF) is characterized by impaired mucus hydration and clearance due to mutations in the *CFTR* gene leading to chronic pulmonary infection and (neutrophilic) inflammation [150]. The sputum of patients with CF is heavily loaded with NETs and NET-related proteins. The activity of NE and the presence of MPO are correlated with disease progression, severity and reduction in lung function [151–154]. Interestingly, it was shown that NE has a higher enzymatic activity within the extracellular DNA of sputum from patients with CF [155].

Despite the seemingly negative influence of neutrophils and NET formation on disease severity in the above-mentioned lung diseases, it is also becoming increasingly clear that it is not the NET formation per se that is responsible for worse disease outcomes but rather an imbalance in NET formation and degradation. It was, for example, shown in a murine model of pathogen-induced lung injury that a complete PAD4 deficiency reduced NET formation and, therefore, lung injury but was counterbalanced by an increased bacterial load and inflammation [148]. Additionally, neutrophils seem to be not only responsible for tissue disruption and early lung damage but also for orchestrating later repair. Here they promote epithelial proliferation and release proteases, needed for the processing of the collagen scar [156].

### NETs in autoimmune diseases

Autoimmunity is defined as loss of self-tolerance, meaning that, cellular or humoral immunity or both, respond against endogenous macromolecules and cells. If this response injures cells or tissues, it is usually referred to as autoimmune disease [157]. Despite their importance in pathogen clearance, NETs contribute to the development and pathogenesis of various autoimmune diseases such as RA, SLE, Anti-neutrophil cytoplasmic antibody-associated vasculitis (AAV), anti-phospholipid Syndrome (APS), psoriasis, and others [158, 159].

Disruption of the balance between NET formation and degradation by DNases in favor of the formation results in accumulation of the released chromatin and the associated proteins into the extracellular matrix, the interstitium and into the lumina of vessels and ducts. Here these released nuclear constituents can serve as sources of autoantigens that may drive the development of autoantibodies and immune complexes, especially if the material carries post-translational modifications like oxidation [160], citrullination [161], carbamylation [161], or neoepitopes generated after proteolytic cleavage [162]. These autoantibodies are directed against a plethora of highly variable disease-specific targets, like double-stranded (ds)DNA in SLE [163], citrullinated proteins in RA [164], anti-lysosome-associated membrane protein 2 and anti-MPO in AAV [165, 166], or phospholipids in APS [167]. These autoantibodies can also alter the persistence and immunogenic potential of NETs themselves. Binding of autoantibodies to the chromatin structures stabilizes NETs and prevents their degradation by DNases [168, 169]. Anti-dsDNA-NET complexes of patients with SLE stimulate type I interferon secretion by mononuclear phagocytes, and NF-κB activity in endothelial cells in an Fc-gamma dependent manner. Thus, they enhance inflammatory immune responses and foster the progression of disease and autoimmunity [170]. NET formation in capillaries and aggNET formation in larger vessels activate endothelial cells and the coagulation cascade, and promote platelet aggregation. Together with the autoantibodies, NETs build immune complexes and foster thrombogenesis. The occlusion of vessels, especially those of the microvascular bed, can precipitate organ damage, and can even be fatal [171].

NETs additionally modulate immune responses through interaction with other immune cells and humoral components; NET-associated proteins, especially histones, serve as damage-associated molecular patterns [161]. NETs activate the inflammasome [172], the complement system via classical, alternative and lectin pathways [81, 165], and the coagulation cascade [171]. The formation of NETs by splenic neutrophils induces immunoglobulin class switch and, thus, can shape B cell responses [173]. In SLE, specific LL37-DNA complexes trigger self-reactive memory B cells for autoantibody production [174]. NETs lower the activation threshold of T cells [175], and directly activate production of type I interferons by plasmacytoid dendritic cells (pDCs), the hallmark cytokines of SLE [176].

A self-reinforcing loop of dysregulated NET formation and inflammation is created, as some NET-mediated responses drive further neutrophil attraction and NET formation [158]. In AAV with microscopic polyangiitis and SLE, this loop is exacerbated by reduced DNase1 activities and the consecutive accumulation and aggregation of NET remnants [177]. Low DNase activities can occur by genetic deficiency [178], consumption of the enzyme, circulating inhibitors [168] [179] or autoantibodies impairing the activity of DNase1L3 as observed in patients with sporadic SLE [180]. Hence, prevention of NET formation by inhibitors or supporting NET degradation by addition of DNases represent therapeutic approaches for the treatment of NET-driven chronic autoimmune diseases [171, 181].

### Obstruction of exocrine ducts and stone diseases

The original defensive function of NETs can contribute to the development of obstructive and subsequently inflammatory diseases when ducts of exocrine glands are affected. Neutrophils physiologically patrol the ducts of exocrine organs [122, 182, 183]. The factors that trigger NET and aggNET formation within the ducts are various, ranging from changes in ion concentrations or pH, to crystal precipitations, bacteria or foreign bodies [93, 122, 182, 184, 185].

Irrespective of the cause, aggNET formation results in reduction of the excretory flow and further accumulation of occlusive material within the NETs. Secretory stasis, which develops gradually, can in turn create an environment in which calculi can form. This process initiates a vicious circle of further obstruction of the ducts, flow rate reduction and inflammation of the adjacent gland, as reported for the pancreas [122, 186], the gall bladder [182], tooth-supporting tissues [187–189], ocular [190, 191] and salivary glands [185].

By incorporating crystals, pathogens, cellular debris and viable immune cells, aggNETs serve as a glue that increasingly condenses the material to facilitate tophus and calculus formation, as observed in gouty arthritis [161, 192], and stone diseases like cholelithiasis [12], and sialolithiasis [185], and meibomian gland disorders.

Gouty arthritis develops as uric acid precipitates in the form of monosodium urate (MSU) crystals in the joints, causing acute inflammation [93, 193]. The crystals are taken up by resident macrophages [194], followed by NALP3 inflammasome activation [195, 196], pro-inflammatory cytokine secretion, and abundant neutrophil recruitment [197, 198]. The latter bind to the crystals and induce NET formation [199]. During this process, the neutrophils release pro-inflammatory mediators, like tumor necrosis factor α (TNFα) [200] and interleukin-6 (IL-6), and the neutrophil attractant CXCL8 as well as the elicitor of neutrophil extravasation, CCL3, and CXCL10, which plays a critical role in oxidative stress induced inflammation [192, 201, 202]. In the presence of high neutrophil counts, the NETs are not sufficiently degraded by DNases. The NETs tend to co-aggregate with the crystals and form tophi that may reach several cm in size. When the pro-inflammatory boost has terminated, the tophus-borne proteases facilitate the resolution of inflammation by degrading certain cytokines and chemokines [93, 197]. In vitro, MSU crystals also induce ROS-dependent NET formation [193]. Cholesterol crystals activate the complement system, and generate C3a and C5a that facilitate further neutrophil influx [203]. In contact with the crystals NETs are generated and promote growth of gall stones. Various kinds of crystals, among other sterile stimuli, are potent inducers of neutrophil activation and NET formation [204].

Likewise, sialoliths are formed in patients with sialadenitis. The presence of leucocytes in saliva, along with high concentrations of bicarbonate ions and calcium-based crystals, trigger NET formation, a step that contributes to the development of sialoliths [185]. The aggregation of NETs then leads to its growth, a common final path in sialolithogenesis. Sialoliths reflect the mechanism of lithogenesis by their layered structure of alternating organic and inorganic components, resulting in an appositional growth of a stone. Once a macroscopic sialolith is formed, salivary gland ducts may be occluded and lose their function [185]; chronic inflammations and autoimmunity may occur. Further candidates for NET-driven pathologies are stones in kidney, pancreas, prostate, as well as calcinosis cutis.

### NETs in the periodontal crevice precipitate periodontitis

The first encounter between neutrophils and dental biofilms occurs within the space delineated by gingival and oral tooth surfaces. This space, referred to as gingival crevice, is filled with a transudate from blood plasma referred to as periodontal crevicular fluid. The latter is characterized by excessive NET formation in periodontitis [187, 189]. The crevice contains abundant outer membrane vesicles originating from the dental biofilm that are endocytosed by crevicular neutrophils. These vesicles orchestrate bacterial colonisation, delivery of virulence factors, and pathogenesis. Bacteria use outer membrane vesicles to modulate the host’s immune response, which eventually allows the bacteria to evade the immunity of the host [205].

When the pre-activated neutrophils that infiltrate the gingival epithelium enter the periodontal crevice [206], they encounter and endocytose a multitude of lipopolysaccharide-filled outer membrane vesicles [205, 207]. These vesicles, containing components of the bacteria’s outer membrane, had been translocated from the early endosomal compartments into the neutrophils’ cytosols, the caspase-4/11/GSDMD signaling pathway is activated and NETs are formed [208–211]. Indeed, caspase-4/11-deficient neutrophils form fewer NETs when compared with wild type controls [208]. Surprisingly, the toll-like receptors (TLR) 2, 3, 4, 7, and 9 are not required for NET formation by neutrophils stimulated with outer membrane vesicles from oral pathogens in vitro [212]. TLR4 is not necessary for caspase-4/11-mediated NET formation [211]. Chloroquine (inhibitor of TLR3, 7, and 9) and oxidized 1-palmitoyl-2-arachidonoyl-sn-glycero-3-phosphocholine (inhibitor of TLR2 and 4) did not affect NET formation when activated with supernatant of oral pathogens in vitro [212]. Virtually all crevicular neutrophils appear to be in some stage of NET formation [187]. Despite the ability of caspase 4/11 to induce NETs independently of NE, MPO and PAD4 [59], NE translocation and H3 citrullination was observed in virtually all crevicular neutrophils [187] and suggests their involvement in crevicular NET formation.

### NET formation in ischemic disease

Many steps of NET formation depend on a sufficient supply with oxygen in tissues [213]. Also, changes of pH in the microenvironment influence neutrophil capabilities of NET formation [184]. Tissue alkalosis favors the release of NETs whereas more acidic conditions lead to reduced NET formation [184]. Commonly, tissue hypoxia is accompanied by acidification of the microenvironment due to metabolic changes.

In human tissues, hypoxia-inducible factor-1α (HIF-1α) regulates cellular responses to low oxygen [214, 215]. Under hypoxia, HIF-1α is stabilized and translocated to the nucleus where it induces the transcription of hypoxia-regulated genes [216]. In neutrophils, translocated nuclear HIF-1α upregulates the transcription of NF-kB, prolongs their viability [216], and promotes degranulation and chemotaxis during hypoxia [217, 218].

In human acute myocardial infarction, an ischemic disease, infiltrating neutrophils show high nuclear HIF-1α content and remain primarily viable [219]. In contrast, neutrophils with low nuclear HIF-1α protein levels form NETs [219]. By staying viable in hypoxic tissue neutrophils remain capable of phagocytosis and clear cell debris as an important step of wound healing [220].

### The role of NETs in cancer

The increased occurrence of NETs in tumor indicates a worse prognosis for cancer patients. NETs are associated with a high histopathological tumor grade, disease progression, metastasis and reduced disease-free and cancer-related survival in various cancer entities [106, 221–223]. The presence of NETs in cancer patients is often indirectly detected through high serum levels of MPO-DNA complexes [222, 224, 225] and to a lesser extent directly in the tumor tissues [105, 106]. A recent study showed that citrullinated NETs in human colon cancer tissues were correlated to the stages 3/4 [106].

The functions of NETs in tumorigenesis have been extensively evaluated in murine models. Interestingly, surgical stress and increased LPS levels after postoperative infections were found to induce NET formation concomitantly with an increased occurrence of metastases [222, 225]. NETs may directly support metastasis formation by trapping tumor cells at the distant site through the coiled-coil domain containing protein 25 (CCDC25), which can bind to NETs and is expressed on certain cancer cells (colorectal, breast, prostate, liver) [223]. Moreover, many key processes involved in cancerogenesis and metastasis are co-regulated by NETs. These processes include the establishment of an immune evasive micromilieu, the activation of dormant tumor cells, tumor cell extravasation, angiogenesis and vascular permeability [226, 227]. Moreover, NETs promote a mesenchymal, pro-metastatic phenotype in breast [228], colorectal [106], gastric [229], and pancreatic cancer [230] cell lines by inducing epithelial-mesenchymal transition (EMT) associated with an increased migration and invasion of the tumor cells. Specifically, the protein content of NETs seems to be necessary for the EMT induction [106]. Altogether, the present knowledge indicates that NETs may be active components in the progression of cancer and putative targets of therapy and prevention.

### NETs in ocular diseases

NETs serve important functions in ocular antimicrobial responses [231, 232]. However, NETs are also involved in pathologies of the eye. Severe cases of chronic Dry Eye Disease (DED) in graft-versus-host disease have been associated with NETs [233] and hyperosmolar stress is thought to induce NET formation on the ocular surface of patients with DED [234]. NETs are involved in molecular pathological alterations in patients with corneal injuries [232, 235]. The choroidal and retinal compartments can also be affected by NETs. Patients with Behcet’s disease, a subtype of non-infectious uveitis, showed an increased formation of NETs possibly responsible for the extended vasculitis in this disease [236]. In vivo and in vitro data of diabetic retinopathy, a major reason for irreversible blindness worldwide, suggest that high blood glucose levels can induce NET formation [237, 238].

## Perspective

In addition to the aforementioned diseases, further moonlighting tasks of extracellular chromatin in the form of NETs come to light. There is increasing evidence that NETs contribute to peritoneal adhesions and traumatic spinal cord injury as discussed shortly in the following paragraphs.

### NETs in adhesions

Peritoneal adhesions are a common consequence of serosal repair after almost all abdominal interventions. Adhesions are associated with serious complications such as intestinal obstruction, pelvic pain, and infertility [239]. As a result, the quality of life of millions of patients throughout the world is affected. In the US, complications from adhesions cost more than two billion dollar per year and are responsible for more than 5% of hospital re-admissions in surgery [240].

Recently, it has been reported that formation and aggregation of NETs worsened primary and secondary intention wound healing. NETs intensified and prolonged the inflammatory phase [241]. Activated neutrophils have been found in burn patients even months after the initiating thermal injury [242]. DNases reportedly accelerated dermal wound healing in mice and in diabetic patients [238, 241]. Contrary to the notion that inflammation is essential for wound healing, areas with low levels of neutrophils, macrophages and T cells, like oral wounds, healed faster with almost no scarring [243].

Despite its clinical impact, the pathomechanisms of adhesions remain poorly understood. Adhesion formation is a form of peritoneal healing which consists of hemostasis, angiogenesis, and tissue remodeling [244]. The most important factor appears to be the inflammation orchestrated by the innate immune system [245]. Within hours, neutrophils are recruited to sites of bacterial infection or sterile tissue injury where they start phagocytosis, degranulation, and NET formation [18, 244].

A recent preprint describes that abundant fibrin-associated NET deposits form murine as well as human adhesions. The digestion of extracellular DNA with DNases abolished the formation of adhesions induced after surgical procedures. These data suggest that NETs form the first scaffold and that fibrin attachment stabilizes the primary structure to eventually form mechanically robust adhesions [246]. Mice with a targeted deletion of PAD4 (*Padi4*^*-/-*^) or mice treated with DNases showed significantly reduced adhesions.

### NETs in spinal trauma

Traumatic spinal cord injury (tSCI) can result in permanent paralysis of patients. Beside individual sequelae and psychosocial trauma, the socioeconomic burden is substantial [89]. A recent multicenter cohort study showed that tSCI patients are among the most resource intense patient group of $11.193 per admission in Canada [247]. Treatment strategies include surgical intervention, type and timing of anticoagulation, as well as the use of corticosteroids. The long-term use of the latter has a number of adverse effects. Evidence-based strategies and novel pharmacologic treatment options are lacking [248–250].

The pathophysiology of tSCI consists of two distinct phases. A primary mechanical injury disrupts axons, neuronal cells, surrounding glia cells, and the blood-brain barrier. Neutrophils accumulate locally within minutes after spinal injury and initiate a phase of secondary damage via the release of NETs, aggravating cell damage and severity of neurological deficits after the initial trauma [251]. Secondary injuries include vascular damage, disturbed hemostasis, edema formation, and particularly inflammation [248].

A murine model showed that a decrease of neutrophil infiltration by selective inhibition of phosphodiesterase 4 (PDE4-I 0.5 or 1.0 mg/kg s.c. bolus; IC486051) decreased MPO, key markers of oxidative stress, and leukocyte infiltration. This resulted in cellular protection, locomotor improvements (by the Basso-Beattie-Bresnahan scale (BBBS)), and reduced neuropathic pain [252]. MPO, a hallmark enzyme of NETs, increased the levels of the pro-inflammatory cytokines IL-6, IL-1ß and TNFα [253]. Indeed, in a murine model of spinal tSCI, the lack of MPO reduced neutrophil infiltration, pro-inflammatory cytokine expression and apoptosis. Consequently, the motor recovery of the MPO knockout mice was improved (by BBBS). The findings indicate that MPO precipitates secondary injury and exacerbates tissue damage after tSCI, mainly via MPO-derived HOCl mediated apoptotic cell death [254].

Interestingly, intravenous DNase1 treatment (5 mg/kg) of rats one hour after tSCI decreased pro-inflammatory cytokine levels in favor of the anti-inflammatory IL-10. The treatment attenuated the NET-induced neuroinflammation and the tSCI-associated edema; it reduced glial and fibrotic scarring as quantified 28 days after injury [251].

**Fig. 3:**
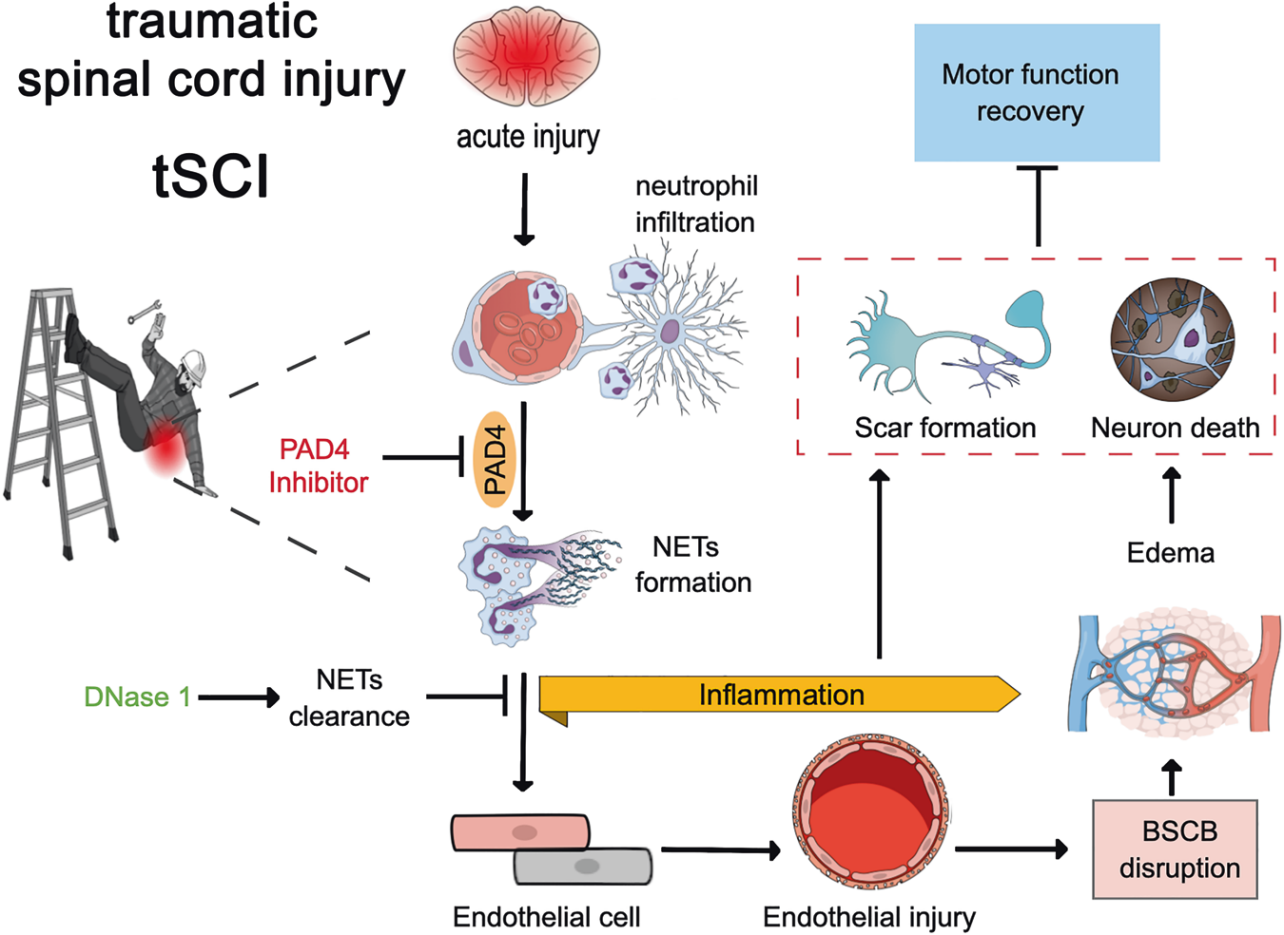
NETs exacerbate secondary injury and promote inflammation in spinal cord injury. Neutrophils infiltrate the lesion core within hours and release NETs which lead to local tissue damage disrupting the blood-spinal cord barrier (BSCB). Resulting tissue hypoxia promotes neuronal apoptosis. Inhibition of peptidylarginine deiminase 4 (PAD4) or degradation of NETs via DNase1 could alleviate damage and promote functional recovery after tSCI.

Considering these findings, anti-NET-therapies (PAD4 or DNase1) should be evaluated in further mechanistic studies (Fig. 3). The aim of these studies is to transfer in vitro data and the pre-clinical findings of the animal models from bench-to-bedside into patients with tSCI. Topical applications of DNases could be greatly beneficial during surgical decompression of the spine, minimizing secondary injury and scarring. Pharmacological stunning of neutrophils during the extended surgical window or treatment of patients with heparin, which has been shown to dismantle NETs and to limit NET formation [103, 255], are further treatment possibilities.

## Conclusion

Chromatin extrusion and the formation of NETs have been the subject of intense investigation since its discovery in 2004. The characteristic feature of these DNA traps is to limit the spread of invading pathogens, kill or suppress them and preserve tissue integrity. NET formation is evolutionarily preserved and must be considered advantageous (Graphical Abstract). Generally, NETs are beneficial. However, there is also a downside to NETs. In their fight against invaders, infiltrating neutrophils and excessive NET formation trigger many pathological processes. NETs can be considered offenders in immunothrombosis, autoimmune diseases, gout, obstruction of exocrine ducts and stone formation, periodontitis, adhesions, spinal trauma, cancer and ocular disorders (Graphical Abstract). Given the involvement of NETs in various pathologies, several state-of-the-art technologies have identified moonlighting extranuclear DNA-protein complexes. As a result, many interventions, especially regarding different forms of DNase and heparin, have been shown to accelerate the disruption and clearance of NETs. However, effective therapies are required for the future that can block pathways leading to the aberrant formation of NETs and preserve the ejected DNA’s protective role.
